# A prospective randomized study in 20 patients undergoing bilateral TKA comparing midline incision to anterolateral incision

**DOI:** 10.1007/s10195-017-0444-0

**Published:** 2017-02-11

**Authors:** Rajesh N. Maniar, Tushar Singhi, Arun Nanivadekar, Parul R. Maniar, Jaivardhan Singh

**Affiliations:** 1 Lilavati Hospital, A-791, Bandra Reclamation, Bandra (W), Mumbai, 400050 Maharashtra India; 2 Department of Orthopedics, Padamshree D Y Patil Medical College, Sector 7; Nerul, Navi Mumbai, 400706 Maharashtra India; 3 51-B, Nook Apartment, S.V. Road, North Avenue Junction, Santacruz (W), Mumbai, 400054 Maharashtra India; 4 Agarwal Ramkrishna Care Hospital, Raipur, 492001 Chattisgarh India; 5 Breach Candy hospital, 60 A, Bhulabhai Desai Road, Mumbai, 400026 Maharashtra India

**Keywords:** Anterolateral incision, Flap numbness, Scar healing

## Abstract

**Background:**

Lateral flap numbness is a known side-effect of midline skin incision in total knee arthroplasty (TKA) and a cause of patient dissatisfaction. Anterolateral incision is an alternative approach which preserves the infrapatellar branches of the saphenous nerve and avoids numbness. Studies have compared both incisions, but in different patients. However, different patients may assess the same sensory deficit dissimilarly, because of individual variations in anatomy and healing responses. We compared the two incisions in the same patient at the same time, using an anterolateral incision on one knee and a midline incision on the other knee in simultaneous bilateral TKA. Other surgical steps including medial arthrotomy were idential. We also correlated subjective and objective findings.

**Materials and methods:**

Twenty patients were prospectively randomized. Sensory loss and skin healing were assessed at 6, 12 and 52 weeks. Subjective preference for the knee with less numbness was charted on Wald’s Sequential Probability Ratio Test. Sensation scores for touch, vibration, static and moving two-point discrimination were measured. Scar healing was evaluated using the Patient and Observer Scar Assessment Scale (POSAS). Functional scores were measured.

**Results:**

A statistically significant difference favoring knees with anterolateral incision was observed in patient preference at all assessment points and this correlated with sensation scores. A statistically significant difference was observed in POSAS score favoring knees with anterolateral incision at 6 and 12 weeks which became statistically insignificant at 1 year. Functional scores remained comparable.

**Conclusion:**

We recommend anterolateral incision as a safe and effective method to circumvent the problem of lateral flap numbness with midline incision.

**Level of evidence:**

I.

## Introduction 

Midline incision is the commonly used approach for total knee arthroplasty (TKA). Lateral flap numbness is a known side-effect resulting from surgical trauma to the infrapatellar branches of the saphenous nerve [12, 22]. Reports in the literature show a varying incidence (55–100%) of postoperative lateral flap numbness at 6 weeks to 8 years [3, 7–9, 19, 20]. This numbness may become permanent [3, 7, 9, 19, 20], leading to dissatisfaction with surgical results [3, 7, 15, 16]. Muller proposed using an anterolateral incision instead, which spared the infrapatellar division of the saphenous nerve [17]. Laffosse et al. [13], in a prospective randomized study reported significantly less area of numbness with anterolateral incision compared to midline incision, when compared in different patients.

Anatomically, an anterolateral incision preserves the blood supply to flaps on either side unlike a midline incision where the blood supply to the lateral flap is compromised [4].

To date, studies in the literature have compared anterolateral versus midline incision on different patients. The anatomy of nerves and blood vessels in individual patients varies, as does the healing of surgical wounds and nerve recovery. Furthermore, the same sensory deficit in different patients may be assessed differently because of individual subjective differences. Comparing the two incisions in the same patient in staged bilateral TKA could again involve variable responses due to the time interval between comparisons. To negate these variable factors, we undertook to compare anterolateral versus midline incision in the same patient undergoing bilateral TKA simultaneously, where medial arthrotomy and all surgical steps remained identical.

Our aims were to (1) identify subjective patient preference with regard to the knee with less postoperative flap numbness, (2) record sensations in the flaps around each incision and see whether they correlate with patient preference, (3) compare wound healing, (4) note scar hyperesthesia, and (5) compare postoperative functional recovery.

## Materials and methods

This was a prospective randomized study. All osteoarthritis patients undergoing simultaneous bilateral TKA by a single surgeon (RNM) between November 2010 and March 2012 at (Lilavati Hospital and Research Centre & Breach Candy Hospital and Research Centre) were given the option of participating. Preoperative assessment was performed after hospital admission. Preoperatively, all sensations were tested and checked to be equal on right and left knees and also equal in the medial and lateral quadrants on either side of the midline over each knee. Patients were excluded if they had (1) a diagnosis other than osteoarthritis, (2) prior trauma or surgery on the knee, (3) peripheral or central neurologic impairment, and (4) peripheral vascular disease. Institutional Review Board clearance from both institutions was given and the study is registered with the Indian Council of Medical Research (registration number 2010/091/001206). Of 30 patients screened, 20 consented as per Council guidelines [6]. Informed consent was obtained from all individual participants included in the study. All consenting patients received the allocated treatment and no patient was lost to follow-up. All patients were female and had a diagnosis of osteoarthritis. The mean age of the patients was 63.3 years (range 52–77 years). All patients had bilateral varus deformity. Mean body mass index (BMI) was 30.5 (range 19.7–40.5). All patients were operated under combined spinal−epidural anesthesia. All patients underwent surgery with a midline incision on one side and an anterolateral incision on the other side. The side receiving anterolateral or midline incision was randomized by a junior resident picking a sealed envelope.

**Fig. 1:**
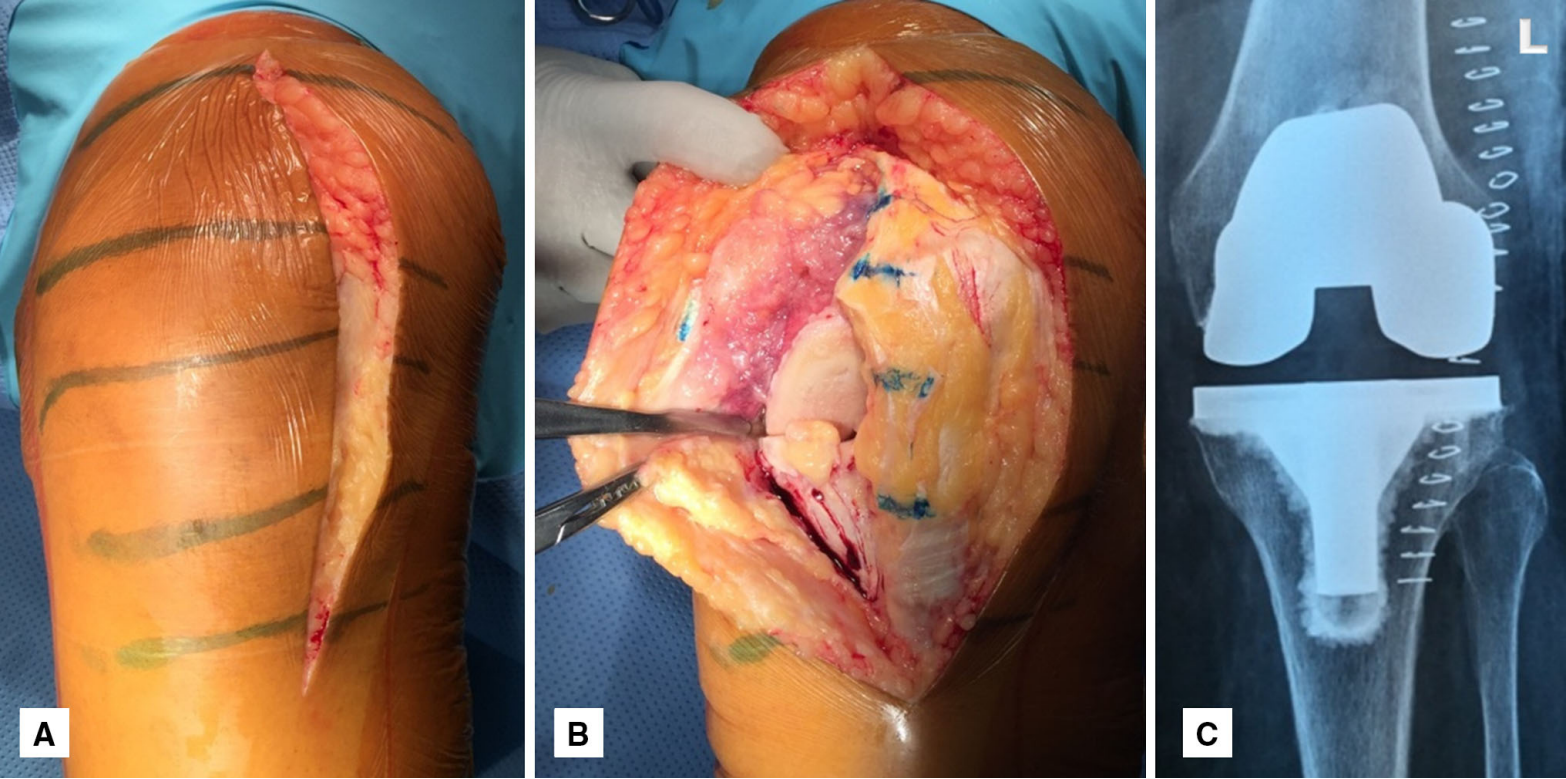
Photograph of a left knee showing **a** anterolateral skin incision, **b** medial arthrotomy and the raised medial flap, and **c** postoperative radiograph of the same patient

The midline incision was started 5–8 cm proximal to the superior border of the patella and extended over the patella in the midline towards the medial aspect of the tibial tuberosity. The anterolateral incision described by Bauer et al. [1], and also used by other authors [13, 18], began in the midline, 5–8 cm proximal to the superior border of the patella, extended anterolaterally, 1 cm lateral to the patella, and then distally, ending just lateral to the tibial tuberosity. The starting point and level at which each incision ended remained the same. The deep fascia was incised in line with both skin incisions. Subfascial dissection was performed to expose for medial arthrotomy (Fig. 1). Except for skin incision, all surgical steps remained identical including medial arthrotomy (Fig. 1). Computer navigation (Kolibri navigation system; Brainlab, Munich, Germany) was used, with array pins placed within the incision for both femur and tibia. It was used to make and verify tibial and femoral cuts and also for balancing. Once alignment was assessed and necessary balancing with trial implants was in place, the navigation arrays were removed. This was followed by preparation for cementing and implantation of final components. All knees were implanted with the same P.F.C.^®^ Sigma^®^ Knee System (DePuy, Warsaw, IN, USA) with resurfacing of the patella. All implants were cemented. Closure was carried out in four layers with Vicryl^®^ no. 1 for deeper layers, Monocryl^®^ 3-0 for subcutaneous closure, and staples for skin closure. Epidural anesthesia was continued for postoperative pain relief for a period of 36–48 h. No local injections or blocks were given for pain relief. Postoperative pain control (using diclofenac, paracetamol, morphine) and rehabilitation protocol remained the same for all patients.

The following parameters were recorded at 6, 12 and 52 weeks postoperatively at the surgeon’s clinic.

1. Subjective patient preference—each patient was asked whether both knees felt similar. If not, he/she was asked to indicate which knee felt better and why. If the reason for preferring one knee over the other was less numbness, it was charted as the preferred knee. If both knees felt the same or if the reason for preferring one knee was other than less numbness, it was not charted and not included.
2. Objective sensation scores—the anterior aspect of each knee was divided into six quadrants, designated as upper medial, upper lateral, middle medial, middle lateral and lower medial, lower lateral quadrants. Each sensation was tested in the middle of each quadrant, approximately 2.5 cm away from the incision. Each medial quadrant was tested first, followed by its corresponding lateral quadrant. Five sensations were tested in these six quadrants.
  - Touch was tested using a Semmes–Weinstein 5.07 monofilament (10 g) whose tip was pressed against the skin until it bent [13].
  - Pain was tested by means of a pin prick; the sharp edge of a sterile pin was pressed on the area to be tested, medial quadrant first, until the patient complained of pain or the skin showed indentation. It was then similarly pressed with equal pressure over the corresponding lateral quadrant for comparison.
  - Vibration was tested using a tuning fork of 256 Hz. With the patient’s eyes closed, a vibrating tuning fork held between the index finger and thumb was placed with its base over the area to be tested. It was similarly applied over the other corresponding quadrant for comparison.
  - Static and moving two-point discrimination were tested using a compass with rounded tips, adjustable for 1-mm increments.
  Scoring—the patient was asked to compare touch, pain and vibration in each lateral quadrant against its medial counterpart. He/she was also asked to categorize it as equal to medial sensation (marked score 0), or less than medial sensation, yet >50% of its judged value (marked score 1) or <50% of the judged medial sensation value (marked score 2). The three scores in the three lateral quadrants were then added. In this manner, sensations of touch, pain and vibration were each scored between minimum 0 and maximum 6.
  For static and moving two-point discrimination, the sensations were measured in millimetres. Each lateral quadrant was compared against its medial counterpart. A score of 0 was given if the lateral quadrant value was equal to its corresponding medial quadrant, a score of 1 was given if the lateral quadrant value was more than medial but less than twice its recorded value, and a score of 2 was given if the lateral quadrant value was more than twice the medial quadrant recorded value. In this manner, both static and moving two-point discrimination were each scored between minimum 0 and maximum 6.
  The total sensation score of each incision was calculated by adding all the scores of touch, pain, vibration, and static and moving two-point discrimination. The total sensation score thus ranged from minimum 0 to maximum 30. The higher the score, the greater was the loss of sensation.
  To determine inter-observer and intra-observer variability in measuring, this score was measured in 10 consecutive TKA patients who were not included in the study. Parameters were recorded at two different times, i.e., 7 days apart by two different evaluators and compared. The 95% limits of agreement and 95% confidence interval of the mean ratio (Table 1), both showed excellent intra-observer and inter-observer agreement.

**Table 1:** Inter-observer and intra-observer variability in measurement of sensation score

S. no./groups to be compared	Sensation score				*p* value	Limits of agreement	Mean ratio	95% confidence interval
	O1S1	O1S2	O2S1	O2S2				
1	16	16	16	16				
2	27	27	27	27				
3	18	19	19	19				
4	20	19	20	19				
5	17	18	19	18				
6	21	22	21	21				
7	18	18	18	18				
8	24	24	23	24				
9	17	17	17	17				
10	25	26	25	26				
O1S1 vs O2S1					0.44	1.99–1.59	0.99	0.96–1.02
O1S2 vs O2S2					0.34	0.62–0.82	1.00	0.99–1.01
O1S1 vs O1S2					0.19	1.81–1.21	0.98	0.96–1.00
O2S1 vs O2S2					1.00	1.51–1.51	1.00	0.98–1.02

*O1S1* observer 1—1st measurement, *O1S2* observer 1—2nd measurement, *O2S1* observer 2—1st measurement, *O2S2* observer 2—2nd measurement, *S.no.* serial number
3. Scar assessment was carried out separately using the Patient Scar Assessment Scale and the Observer Scar Assessment Scale. The two were individually scored and added to obtain a combined Patient and Observer Scar Assessment Scale (POSAS) score out of 110 [5, 23].
4. Postoperative scar hyperesthesia was noted, if present.
5. A function subscore of the Knee Society Score (KSS, Western Ontario and McMaster Universities Osteoarthritis Index (WOMAC), and 12-Item Short Form Health Survey (SF-12) were recorded for each patient. Pain on the VAS scale and range of motion (ROM) and KSS knee subscore were recorded for each knee. Duration of surgery beginning from skin incision to completion of wound closure was noted.

The above parameters were subjected to statistical analyses as follows.

1. Subjective patient preference was analyzed by Wald’s Sequential Probability Ratio Test (SPRT). Charts based on Wald’s SPRT were generated using True Epistat, version 5.3 (Epistat Services, Richardson, TX, USA, 1995). Preference for the knee with anterolateral incision was marked as a one-unit line in a north-east direction, preference for the knee with midline incision was marked as a one-unit line in an east direction, and no preference amongst the two knees was not marked, i.e., tied pairs were not included (Fig. 2). The order of charting preference followed the accrual and operation dates of the patients.

**Fig. 2:**
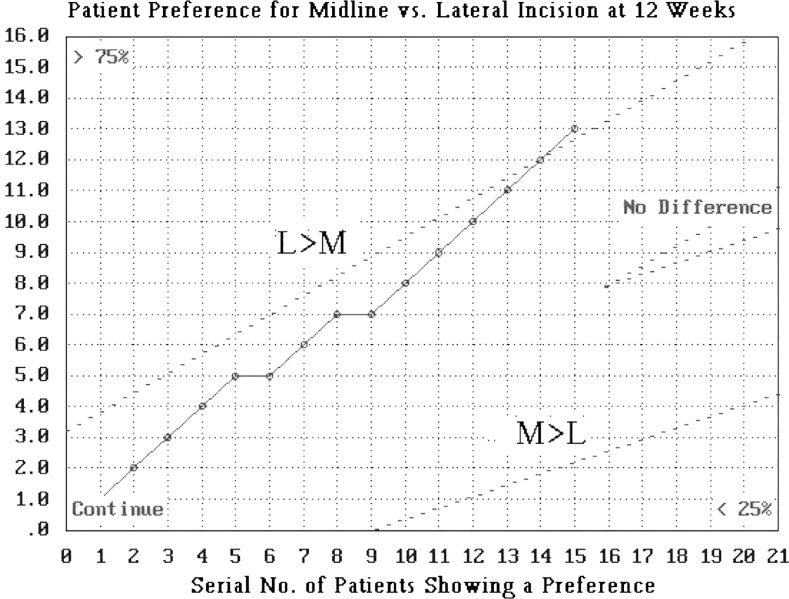
Wald’s sequential probability ratio test charting patient preference at 12 weeks after surgery
  The charts generated were examined to see which boundary was crossed. If the upper boundary was crossed, it implied that knees with anterolateral incision were preferred by the patients. If the lower boundary was crossed, it implied that knees with midline incision were preferred. If none of the two boundaries were crossed, it implied that patients did not prefer any one knee over the other. This method allowed us to conclude the trial as soon as the boundary was crossed. The upper boundary was crossed after charting the preferences of 19 patients; however, at that point the last patient was already recruited and so was allowed to complete the trial.
2. Sensation scores were compared by paired *t* test. Their results were compared with Wald’s SPRT results of patient preference to see whether both correlated.
3. POSAS scores were compared by McNemar’s paired chi-squared test.
4. WOMAC, SF12 and the KSS function subscore for each patient remained the same for both incisions. The KSS knee subscore, ROM and pain on the VAS scale were compared between the knees with the two incisions by paired *t* test.

## Results

1. Subjective patient preference charted by Wald’s SPRT showed that no boundary was crossed at 6 weeks, indicating that none of the two knees with either incision was preferred. At 12 weeks, the upper boundary was crossed, implying that knees with an anterolateral incision were preferred (Fig. 2). This same preference was maintained at 52 weeks.
2. Paired *t* test evaluation of sensation scores (Table 2) revealed significantly lower scores for knees with anterolateral incision compared to midline incision at each follow-up. This implied significantly less loss of sensation in the lateral flaps of knees with an anterolateral incision.

**Table 2:** Comparison of sensation score between midline and anterolateral incisions

Time of assessment	Type of incision	No. of patients	Sensation score mean (SD)	*p* value*
6 weeks	Anterolateral	20	11.65 (4.08)	<0.001^#^
	Midline	20	17.25 (3.84)	
3 months	Anterolateral	20	9.4 (5.04)	0.0002^#^
	Midline	20	14.75 (5.25)	
1 year	Anterolateral	20	1.15 (1.90)	0.006^#^
	Midline	20	3.85 (4.1)	

* Statistical evaluation by paired *t* test; significant if <0.05

^#^ Significant difference
  Furthermore, the sensation scores with both incisions kept decreasing in value from 6 to 52 weeks postoperatively. This implied that numbness reduced over time with both incisions.
  Although recovery of sensation was seen with both incisions, the final recovery at 52 weeks in knees with anterolateral incision was significantly better (*p* = 0.0060) than in knees with midline incision (Table 2).
  Results of static and moving two-point discrimination revealed no difference between the preoperative and postoperative values over the medial flap in both anterolateral and midline incisions at all assessment points. On the other hand, static and moving two-point discrimination values over the lateral flap with both incisions were higher at 6 weeks postoperatively compared to preoperative values, regardless of which incision was used. These values, like other sensory parameters continued to improve until 52 weeks.
  Overall, the objectively measured sensation scores correlated with subjective Wald’s SPRT.

3. POSAS scores, when evaluated, showed that they were lower for knees with an anterolateral incision, implying better healing with the anterolateral incision at 6 and 12 weeks, compared to midline incision. However, at 52 weeks, they became comparable (Table 3) (Fig. 3).

**Table 3:** Comparison of scar healing (POSAS score) between midline and anterolateral incisions

Time of assessment	Type of incision	No. of patients	POSAS^#^ mean (SD)	*p* value*
6 weeks	Anterolateral	20	23.15 (6.71)	0.0019^$^
	Midline	20	28.9 (10.46)	
3 months	Anterolateral	20	18.1 (7.14)	0.0103^$^
	Midline	20	22.7 (5.92)	
1 year	Anterolateral	20	16.8 (7.05)	0.4218
	Midline	20	17.5 (6.6)	

^#^ Lower score implies better scar healing

* Statistical evaluation by paired *t* test; significant if <0.05

^$^ Significant difference

**Fig. 3:**
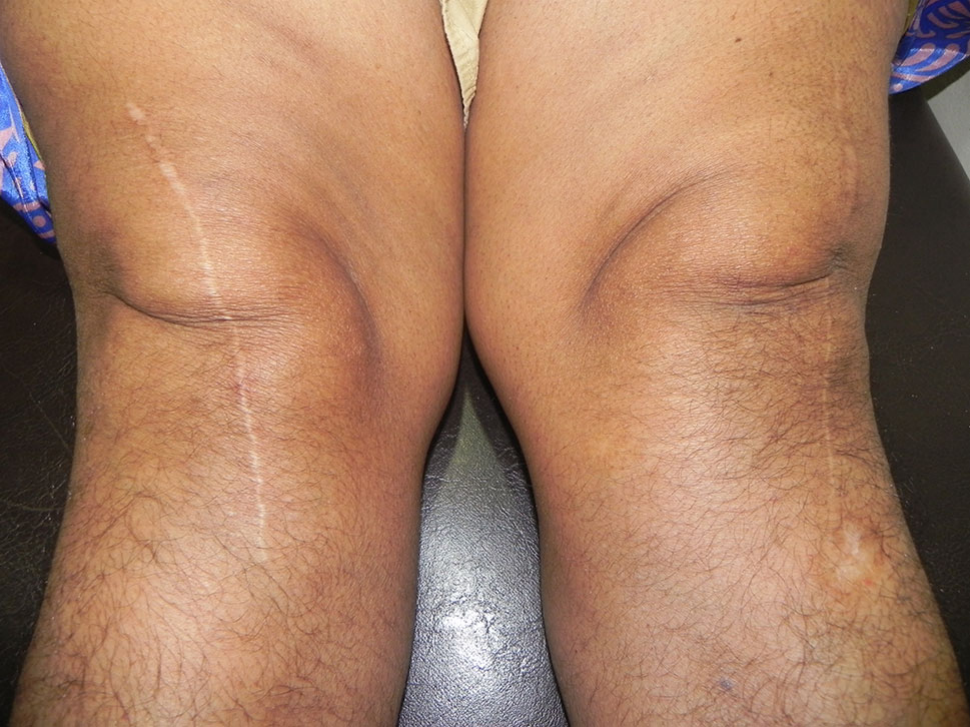
Clinical photograph at 52 weeks postoperatively, showing a right knee midline scar and a left knee anterolateral scar
4. Scar hyperesthesia—5 patients recorded lateral flap hyperesthesia in knees with both midline and anterolateral incision and 5 patients recorded lateral flap hyperesthesia only in knees with midline incision. No patient complained of medial flap hyperesthesia in any knee.
5. Average SF-12 physical component score (PCS) improved from 30 (21–55) preoperatively to 46 (30–80) at 12 weeks and 46 (33–56) at 52 weeks postoperatively. Average SF-12 mental component score (MCS) improved from 51 (40–71) preoperatively to 54 (32–70) at 12 weeks and to 54 (18–64) at 52 weeks postoperatively. Average WOMAC score improved from 58 preoperatively to 26 at 12 weeks and 13 at 52 weeks postoperatively. Average KSS increased from 88 (28–130) preoperatively to 163 (71–200) at 12 weeks and 184 (80–200) at 52 weeks postoperatively. Average KSS knee subscore for midline incision improved from 37 (8–72) preoperatively to 90 (28–100) at 12 weeks and 100 (96–100) at 52 weeks postoperatively. Average KSS knee subscore for anterolateral incision improved from 37 (10–66) preoperatively to 90 (28–100) at 12 weeks and 100 (93–100) at 52 weeks postoperatively. Statistical analysis showed no significant difference in KSS knee subscore, ROM and pain on the VAS scale between the knees at all assessment points.

There were no intra-operative complications. One patient with midline incision had wound dehiscence (1 inch) at the proximal end of the incision on day 14 after surgery when skin clips were removed. The wound healed after re-application of skin stitches. No patient had postoperative superficial or deep infection, necrosis of skin flap or neuroma formation.

The average duration of surgery with midline incision was 133.9 min (117–155 min) and with anterolateral incision was 137.5 min (117–160 min). The difference of 3.8 min was too small to be of any significance clinically.

## Discussion

Our study was primarily aimed at identifying patient preference between knees with anterolateral and midline skin incisions in terms of reduced postoperative lateral flap numbness under identical surgical conditions. Our secondary aims were to compare sensation scores, scar healing, other functional parameters and the presence or absence of scar hyperesthesia.

Limitations are (1) six quadrants of the anterior knee were tested, but the extent of the area over which the numbness existed was not measured. Extent of numbness has been studied previously [13], but different sensations were not quantified as in our study. (2) Spectrum of sensation testing should include hypoesthesia, paraesthesia, dysesthesia and hyperesthesia. We measured hypoesthesia and noted the presence of hyperesthesia, if present. (3) All our patients had varus deformity. Thus, we have been unable to correlate results with the type of deformity. In spite of these limitations, our study is noteworthy because the two incisions were compared in the same patient at the same time, thus eliminating all individual-related and time-related variables. Moreover, except for the skin incisions, arthrotomy and all other steps of surgery remained identical for ideal comparison. Lastly, subjective and objective comparisons were made and correlated.

Subjective patient preference for the knee with less lateral flap numbness and the objective sensation scores in our study were both in favor of anterolateral incision at 12 weeks and at 52 weeks. Our results are similar to those reported by Berg and Mjoberg [2] and Laffosse et al. [13] and explained by greater preservation of the infrapatellar branches of the saphenous nerve in anterolateral skin incision. Follow-up scores showed that sensations recovered with time in both incisions but the recovery was significantly better with anterolateral incision even at 1 year post-surgery. Laffosse et al. showed that with time, the area of lateral flap numbness becomes smaller [13]. Our study further shows that the quality of sensation within the affected area (pain, touch, vibration, two-point discrimination) also improves with time [1, 4, 13, 18].

Scar assessment as judged by the POSAS score signified better healing with anterolateral incision at 6 and 12 weeks but no difference at 52 weeks. Blood supply to the anterior knee comes predominantly from the medial side. A watershed area exists on the anterolateral aspect of the knee where circulation from the medial and lateral sides meet [4]. A midline incision would create a lateral skin flap whose medial portion (medial to the watershed zone) has its blood supply compromised. An anterolateral incision would create a lateral flap whose blood supply is not thus compromised. Previous studies have shown reduced blood supply to the lateral flap compared to the medial flap when using a midline incision [10, 11]. Furthermore, on bending the knee, an anterolateral incision causes less stretching of the lateral flap, thereby maintaining better blood flow [4, 14]. Shetty and Shetty [18] have similarly reported early and better wound healing with an anterolateral incision in their comparative study. In our study, we observed no difference in the scars between the two incisions at 1 year post-surgery.

The average duration of surgery with anterolateral incision was 3–4 min more than with midline incision. Medial arthrotomy with anterolateral incision involved raising the medial flap and closing as a curved incision which entailed extra minutes of surgical time. This difference was clinically insignificant and may become negligible when anterolateral incision is routinely used.

If revision surgery becomes necessary in future, the anterolateral incision can be reused by raising the medial flap and performing medial arthrotomy. The incision could be extended proximally or distally, as needed. We have not yet had an opportunity to do so.

The few studies in the literature which have compared midline, medial parapatellar and anterolateral incisions in terms of lateral flap numbness are summarized in Table 4. Midline and medial parapatellar incisions have been shown to be equal in this respect, whereas anterolateral incision was always shown to be better. Tanavalee et al. [21] recently reported a comparative study between the standard medial parapatellar incision and a minimally invasive medial parapatellar incision for the resulting area of numbness and reported no difference.

**Table 4:** Summary of studies comparing midline, medial parapatellar and anterolateral incisions

Study	Type of study	Variables in comparison	Type of incision /no. of knees	Follow-up duration (mean)	Difference in postoperative lateral flap numbness	Postoperative neuroma (no. of years from surgery)	Scar Healing	Wound dehiscence/hematoma formation	Range of motion	Functional recovery
Sundaram et al. [20]	Prospective randomized	Different patients/different time	ML/76	2.7 years	No difference	–	No difference	–	–	–
			MP/91			–		–	–	–
Shetty et al. [18]	Prospective randomized	Different patients/different time	ML/25	6 weeks	Significantly less with AL	–	–	More wound dehiscence with ML/–	Better flexion in AL –	AL better in early flexion recovery –
			AL/24			–				
Berg et al. [2]	Retrospective	Different patients/different time	MP/33	3 years	Significantly less with AL	14/32 (6)	–	–	–	–
			AL/31			3/31 (6)				
Laffose et al. [13]	Prospective randomized	Different patients/different time	ML/32	1 year	Significantly less with AL	0 (1)	–	0/0	No difference	No difference
			AL/31			0 (1)		0/2	–	–
Current study	Prospective randomized in bilateral simultaneous TKR patients	Same patients/same time	ML/20	1 year	Significantly less with AL	0 (1)	No difference at 1 year	1/0	No difference	No difference
			AL/20			0 (1)		0/0		

*ML* midline incision, *MP* medial parapatellar, *AL* anterolateral

We conclude that an anterolateral skin incision with medial arthrotomy in TKA can be safely used and is of value in circumventing the problem of lateral flap numbness post TKA. Subjective numbness and measured sensation scores are better with anterolateral incision at all time intervals up to 1 year. Scar healing is quicker with the anterolateral incision, but the final quality of the scar at 1 year is equal.

## References

[1] Bauer R, Kerschbaumer F, Poisel S (1988). Voies d’abord en chirurgie orthopédique et traumatologique.

[2] Berg P, Mjoberg B (1991). A lateral skin incision reduces peripatellar dysaesthesia after knee surgery. J Bone Joint Surg Br.

[3] Borley NR, Edwards D, Villar RN (1995). Lateral skin flap numbness after total knee arthroplasty. J Arthroplasty.

[4] DePeretti F, Argenson C, Beracassat R, Bourgeon Y. Problèmes artériels et nerveux posés par les incisions cutanées antérieures au niveau de l’articulation du genou (in French) (1987) [Arterial and neurologic problems posed by anterior cutaneous incisions at the level of the knee joint. Rev Chir Orthop Reparatrice Appar Mot73 (suppl 2):231–233 3432683

[5] DraaijersLieneke J, TempelmanFenike RH, Botman Yvonne AM, TuinebreijerWim E, Middelkoop Esther, Kreis Robert W, van Zuijlen Paul PM (2004). The patient and observer scar assessment scale: a reliable and feasible tool for scar evaluation. Plast Reconstr Surg.

[6] Ethical Guidelines for Biomedical Research on Human Participants. Indian Council of Medical Research (2006) Ch. 2, pp 21–33. http://www.icmr.nic.in/ethical_guidelines.pdf

[7] Hopton BP, Tommichan MC, Howell FR (2004). Reducing lateral skin flap numbness after total knee arthroplasty. Knee.

[8] Jariwala A, Kiran M, Parthasarathy A, Johnston L (2015). Numbness around the total knee arthroplasty surgical scar: its prevalence and effect on outcome. Bone Joint J.

[9] Johnson DF, Love DT, Love BR, Lester DK (2000). Dermal hypoesthesia after total knee arthroplasty. Am J Orthop.

[10] Johnson DP, Eastwood DM, Bader DL (1991). Biomechanical factors in wound healing following knee arthroplasty. J Med Eng Technol.

[11] Johnson DP (1988). Midline or parapatellar incision for knee arthroplasty: a comparative study of wound viability. J Bone Joint Surg Br.

[12] Kerver ALA, Leliveld MS, den Hartog D, Verhofstad MHJ, Kleinrensink GJ (2013). The surgical anatomy of the infrapatellar branch of the saphenous nerve in relation to incisions for anteromedial knee surgery. J Bone Joint Surg Am.

[13] Laffosse JM, Potapov A, Malo M, Lavigne M, Vendittoli P-A (2011). Hypesthesia after anterolateral versus midline skin incision in TKA. A randomized study. Clin Orthop Relat Res.

[14] Langrana NA, Alexander H, Strauchler I, Mehta A, Ricci J (1983). Effect of mechanical load in wound healing. Ann Plast Surg.

[15] Mistry D, O’Meeghan C (2005). Fate of the infrapatellar branch of the saphenous nerve post total knee arthroplasty. ANZ J Surg.

[16] Hassaballa Mo, Artz Neil, Weale Adrian, Porteous Andrew (2012). Alteration in skin sensation following knee arthroplasty and its impact on kneeling ability: a comparison of three common surgical incisions. Knee Surg Sports Traumatol Arthrosc.

[17] Muller W (1983). The knee: form, function and ligament reconstruction.

[18] Shetty VD, Shetty GM (2009). Anterolateral incision in total knee arthroplasty: is there a role for a longer incision in this day and age of minimal invasive surgery?. Eur J Orthop Surg Traumatol.

[19] Subramanian S, Lateef H, Massraf A (2009). Cutaneous sensory loss following primary total knee arthroplasty. A two years follow-up study. Acta Orthop Belg.

[20] Sundaram RO, Ramakrishnan M, Harvey RA, Parkinson RW (2007). Comparison of scars and resulting hypoaesthesia between the medial parapatellar and midline skin incisions in total knee arthroplasty. Knee.

[21] Tanavalee A (2016). Area of skin numbness after total knee arthroplasty: does minimally invasive approach make any difference from standard approach?. J Arthroplast.

[22] Tennent TD, Birch NC, Holmes MJ, Birch R, Goddard NJ (1998). Knee pain and the infrapatellar branch of the saphenous nerve. J R Soc Med.

[23] van de Kar AL, Corion LU, Smeulders MJ, Draaijers LJ, van der Horst CM, van Zuijlen PP (2005). Reliable and feasible evaluation of linear scars by the patient and observer scar assessment scale. Plast Reconstr Surg.

